# INDIGENOUS CULTURAL GENERATIVE ACTS TO REDUCE GENERATIVE MISMATCH AND IMPROVE HEALTH OF ALL GENERATIONS

**DOI:** 10.1093/geroni/igad104.0515

**Published:** 2023-12-21

**Authors:** Jordan Lewis

**Affiliations:** University of Minnesota Medical School, Duluth campus, Duluth, Minnesota, United States

## Abstract

The gerontological literature predominantly focuses on aging-related losses and less on the gifts we acquire as we age. Alaska Native Elders have experienced a lifetime of adversity, which persists today, but they have also remained resilient. One characteristics of successful aging among Alaska Native Elders is their commitment and passion for sharing their teachings with the youth. Generativity is concerned with using personal resources to improve the quality of life for future generations. While not commonly used in gerontological social work research, it is a cultural practice among Indigenous Elders. This decade long study has been exploring the concept of successful aging from an Alaska Native perspective, or what it means to age in a good way in Alaska Native communities. Qualitative, in-depth, interviews have been conducted with 154 Elders representing 20 participating communities across the State of Alaska to explore the concept of successful aging and the role of generativity in the aging process. For this presentation, 108 interviews with Alaska Native Elders explored successful aging. This presentation will highlight the critical role generativity plays in Alaska Native Elders’ ability age in a good way, how generativity can be adapted to bridge the generative mismatch happening between generations and support each generation to healthy and meaningful lives. This presentation will also explore innovative and culturally responsive ways to teach the youth about aging in a good way and how families and communities can support their Elders to be meaningful engaged in the rapidly changing families in the Arctic.

